# Modeling Aboveground Biomass in Hulunber Grassland Ecosystem by Using Unmanned Aerial Vehicle Discrete Lidar

**DOI:** 10.3390/s17010180

**Published:** 2017-01-19

**Authors:** Dongliang Wang, Xiaoping Xin, Quanqin Shao, Matthew Brolly, Zhiliang Zhu, Jin Chen

**Affiliations:** 1 National Hulunber Grassland Ecosystem Observation and Research Station, Institute of Agricultural Resources and Regional Planning, Chinese Academy of Agricultural Sciences, Beijing 100081, China; wangdongliang@caas.cn; 2 Key Laboratory of Land Surface Pattern and Simulation, Institute of Geographic Sciences and Natural Resources Research, Chinese Academy of Science, Beijing 100101, China; shaoqq@igsnrr.ac.cn; 3 School of Environment and Technology, University of Brighton, Brighton BN2 4GJ, UK; M.Brolly@brighton.ac.uk; 4 U.S. Geological Survey, Reston, VA 20192, USA; zzhu@usgs.gov; 5 State Key Laboratory of Earth Surface Processes and Resource Ecology, Beijing Normal University, Beijing 100875, China; chenjin@bnu.edu.cn

**Keywords:** UAV lidar, grasslands, canopy height, fractional cover, aboveground biomass

## Abstract

Accurate canopy structure datasets, including canopy height and fractional cover, are required to monitor aboveground biomass as well as to provide validation data for satellite remote sensing products. In this study, the ability of an unmanned aerial vehicle (UAV) discrete light detection and ranging (lidar) was investigated for modeling both the canopy height and fractional cover in Hulunber grassland ecosystem. The extracted mean canopy height, maximum canopy height, and fractional cover were used to estimate the aboveground biomass. The influences of flight height on lidar estimates were also analyzed. The main findings are: (1) the lidar-derived mean canopy height is the most reasonable predictor of aboveground biomass (*R*^2^ = 0.340, root-mean-square error (RMSE) = 81.89 g·m^−2^, and relative error of 14.1%). The improvement of multiple regressions to the *R*^2^ and RMSE values is unobvious when adding fractional cover in the regression since the correlation between mean canopy height and fractional cover is high; (2) Flight height has a pronounced effect on the derived fractional cover and details of the lidar data, but the effect is insignificant on the derived canopy height when the flight height is within the range (<100 m). These findings are helpful for modeling stable regressions to estimate grassland biomass using lidar returns.

## 1. Introduction

Grasslands cover approximately 27% of the earth’s surface with important ecosystem services, such as livestock forage, carbon storage, and soil protection [1,2,3,4]. Aboveground biomass is the most important index of grassland sensitive to climate change and human activity, and is a significant concern among scientists and grassland managers, who require timely and quantitatively measured methods to monitor impacts over short and long temporal periods [5]. Vegetation structure data, such as canopy height and coverage, are important indicators of vegetation health, and can be used to estimate aboveground biomass. Aboveground biomass in turn can be used to determine total available forage [2,6], carbon storage [1], and wind erosion potential for arid and semi-arid lands [7]. Data sources and methods for monitoring canopy height, fractional cover, and aboveground biomass can be summarized by the four following categories: ground-based methods, optical remote sensing-based methods, Synthetic Aperture Radar (SAR)-based methods, and light detection and ranging (lidar)-based methods.

Traditional methods for monitoring biophysical parameters, such as canopy height, fractional canopy cover, and biomass, mainly rely on field measurements from thousands of sampling locations [8,9]. Field-measured data is accurate, but laborious and costly [10,11,12]. Much of grassland is remotely located, often being difficult to access on the ground. The limited number of field measurements obtainable also restricts the accuracy of monitoring biophysical parameters in larger areas.

Optical remote sensing technologies are an increasingly popular source of data capture and visualization tool for monitoring grassland biophysical parameters, such as fractional cover [13], biomass and vegetation growth [2,14,15]. Vegetation indices (VIs) calculated from optical imagery are the most widely used variables [16,17,18,19]. However, the accuracy of estimating canopy height and biomass in grassland ecosystems by spaceborne or airborne remote sensing is varied. Variations are exhibited according to choices of spectral band, investigation season, targeted species, and vegetation water content [2,20]. The saturation problem of VIs, characterized by the lower sensitivity of VIs calculated from various spectral data in response to biomass accumulation when canopy cover exceeds certain thresholds, also severely affects biomass estimation accuracy in grasslands with high canopy cover [21]. Canopy heights can also be extracted by photogrammetric approaches, i.e., by subtracting digital surface models (DSMs) from digital elevation models (DEMs) created from sets of overlapping (i.e., stereo) aerial photographs [20]. The photogrammetric literature has mainly focused on extracting the heights of high vegetation, such as forests and shrubs, using spaceborne or airborne high-resolution imagery [20,22]. The forest canopy height map produced using the Panchromatic Remote-sensing Instrument for Stereo Mapping (PRISM), aboard the Japanese Space Agency’s (JAXA) Advanced Land Observation Satellite (ALOS) was reported to have an *R*^2^ correlation with the RH50 product of the Land Vegetation and Ice Sensor (LVIS) of 0.74, and an RMSE (root-mean-square error) of 2.56 m [22]. The shrub mean heights derived from aerial imagery, 2.3 cm ground resolution, were reported to have a moderate linear relationship with those measured in the field. An *R*^2^ correlation of 0.40 (RMSE of 0.22 m) was reported [20]. The cost of photogrammetric data is relatively low in comparison to lidar, but the accuracy of this method is low for estimating grassland canopy heights. Grasses in arid and semi-arid environments are generally only 10–50 cm tall, which is much lower that the shrubs or forests. Very high-resolution images, of the accuracy required for this purpose (e.g., ground sampling distance <5 cm), are difficult to capture and process.

SAR is a promising/successful technique to estimate canopy height and biomass. Three distinguishable approaches of mapping canopy height and biomass from SAR are approaches based on backscatter, coherence, and phase. Coherence-based approaches rely on the estimation of the complex correlation coefficient between two SAR acquisitions [23,24]. Phase-based approaches, using Interferometric SAR (InSAR) techniques, exploit the interference patterns (so-called fringes) of two SAR acquisitions. Phase differences resulting from the path differences between the two acquisitions are used to estimate topographic height of the scattering phase center [25,26]. Dual wavelength InSAR data or single wavelength Polarimetric Interferometric SAR (PolInSAR) data can be used to estimate canopy heights based on the different vertical locations of the scattering phase centers. For example, higher frequency SAR will produce phase centers higher in the canopy due to sensitivity to smaller scatters and greater attenuation. These approaches has not been reported in grassland ecosystems partly because of the low biomass of grasslands. The third distinguishable SAR method is the backscatter based approach. This approach uses the relationship between the SAR backscattering coefficient and vegetation structure to estimate the canopy height and biomass [27]. This method has been used more widely in the detection of grassland swath events using high frequencies such as X Band [28,29], classification [30], and height and biomass mapping [17]. However, the functional relationship between backscatter and biomass depends heavily on vegetation structure, wavelength, incidence angle, and soil water content [2,20].

Lidar data is widely regarded as the best dataset for estimating both canopy height and fractional cover according to the decomposition of the returns from vegetation and ground [5,31,32]. The derived canopy height and fractional cover have been used to accurately estimate aboveground biomass even in those high biomass ecosystems where passive optical and active microwave sensors typically suffer the saturation problem [33,34,35,36]. The ability to acquire lidar data on multiple scales is also a crucial factor. To date, spaceborne applications of lidars have largely used waveforms from the Geoscience Laser Altimeter System (GLAS) on the Ice Cloud and Land Elevation Satellite (ICESat) developed by the National Aeronautics and Space Administration (NASA). The footprint of this full waveform system is approximately 65 m in diameter while the footprint of the airborne LVIS system is closer to 25 m. Different methodologies have been used to determine vegetation heights using full waveform lidar products as well as those using the more typical approach of discrete return lidar. Lefsky and Michael [37] used the full waveform extent as well as the 10th and 90th percentiles of waveform energy to estimate the 90th percentile patch forest canopy height (*R*^2^ = 0.67 and RMSE = 5.9 m). Gwenzi and Lefsky [33] used the width, lead, and trail of waveforms to model the maximum forest canopy heights, but the accuracy (RMSE) of modeling forest canopy heights was low (*R*^2^ = 0.73; RMSE = 2.53 m). Accuracies were slightly improved when normalized difference vegetation index (NDVI) was included. Dubayah et al. [38] estimated the maximum tropical forest canopy height and biomass using the LVIS system in La Selva, Costa Rica. The maximum canopy heights derived from LVIS were highly correlated with those obtained by a discrete return small-footprint system, with *R*^2^ = 0.97 and RMSE = 3.22 m. Rogers et al. [5] investigated the ability of full-waveform lidar to estimate salt marsh vegetation biophysical parameters, such as canopy height, stem density, and biomass. It was found that the waveform amplitude and waveform standard deviation accounted for nearly 75% of the vegetation height variability. While spaceborne lidar data is limited in terms of footprint separation (point sampling data) it offers the highest level of spatial coverage and as such reduces economic costs over large areas. However, terrestrial or airborne lidar is often too expensive for similarly broad-scale data collection [32,39]. A waveform lidar sensor is typically much more expensive than a common optical camera, while the cost of an airborne survey using discrete return is similarly prohibitive. In addition, it is difficult to install the heavy waveform systems on light unmanned aerial vehicles (UAVs) which would go some way to reduce costs and associated problems relating to continuous coverage commonly associated with spaceborne and airborne systems. Until economically viable wide swath imaging lidar systems and their data are more widely available, these transect lidar data, such as from GLAS, must be combined with optical or microwave images to extrapolate from individual lidar observations to provide a complete horizontal coverage [37]. Such practices will be required until wall-to-wall coverage is available. In addition, lidar-derived canopy heights, where large footprints are used, are reported to underestimate for many species, including those of forests and shrubs [33], as a result of their large footprint size. These disadvantages severely limit current lidar applicability in grassland ecosystems.

In recent years, the emergence of UAVs and smart discrete lidar has paved the way for new applications in environment monitoring. Owing to their low weight and cost, discrete lidar technology is increasingly being used for UAV applications, including for individual tree detection [40] and 3D mapping of forest canopy structural properties [41]. As an example, the Velodyne HDL-32E (Velodyne LiDAR, Inc., Morgan Hill, CA, USA) weighs only 1 kg. In this study, this system was used to test the ability of UAV discrete lidar to model canopy height, fractional cover, and biomass in the Hulunber grassland. We will investigate if the canopy height, fraction cover, and aboveground biomass can be derived using models established from UAV-based discrete lidar data with desirable accuracy at quadrat and subplot scales. We will also determine the influence of the flight height on the accuracy of the models. Canopy height, fraction cover, and aboveground biomass maps at 1.0 m pixel sizes were produced from the UAV-based discrete lidar data acquired in 2015. The accuracy and uncertainty of the prediction models were also analyzed in this study.

## 2. Data and Methods

**Figure 1:**
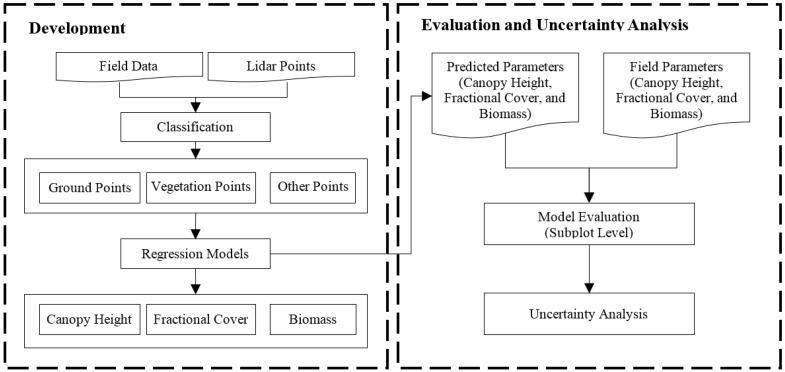
Workflow for model development, evaluation, and uncertainty analysis for mapping canopy height, fractional cover, and biomass maps based on lidar data.

The canopy height and fraction cover indices were first extracted from the UAV-based discrete lidar point data. Linear and nonlinear regression analyses were then carried out between the lidar-derived canopy structure indices and the field measurements. Subsequently, the canopy height, fraction cover, and aboveground biomass were mapped spatially across the study area using lidar-derived indices. Next, we evaluated the prediction models using the stocking rate patterns of the grazing platform at subplot scale. Finally, we analyzed the influence of the flight height on the accuracy of the models and discussed the limitations and applications of the technique in broad-scale grassland ecosystem monitoring. The workflow is illustrated in Figure 1.

### 2.1. Study Area

**Figure 2:**
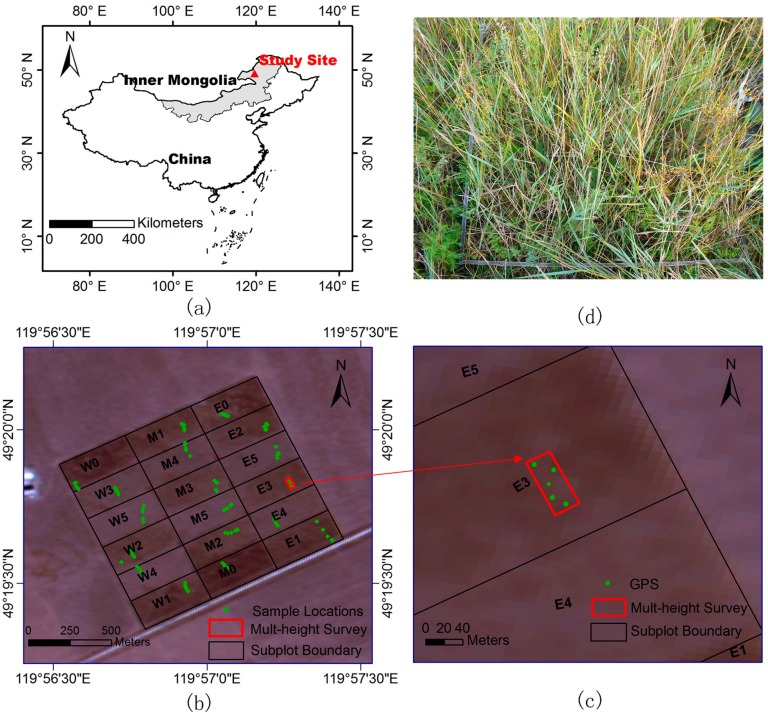
(**a**) Location of Inner Mongolia, China and the study site in Inner Mongolia; (**b**) cattle-grazing plot comprising 18 canopy subplots. W0 to W5, M0 to M5, and E0 to E5 indicate West, Middle, and East replicates of the six stocking rates (0–5); (**c**) location of mult-height survey; and (**d**) one of the randomly selected quadrats.

This research was conducted in the cattle-grazing plot of Hulunber Grassland Ecosystem Observation and Research Station (HGEORS), located at the center of the Hulunber meadow steppe in the northeastern region of Inner Mongolia, China (49°19′349′′ N, 119°56′521′′ E, Figure 2). The cattle-grazing plot has an area of 1 km^2^. Across the plot, elevation varies from 666 m to 680 m. The overall terrain slope across the plot is less than 3°. The climate is temperate semi-arid continental, with an annual average of 110 frost-free days. Annual mean precipitation ranges from 350 mm to 400 mm, approximately 80% of which falls between July and September. The vegetation is characterized as typical meadow steppe. The dominant species include *Leymus chinensis*, *Stipa baicalensis*, *Carex duriuscula*, *Galium verum*, *Pulsatilla turczaninovii*, *Bupleurum scorzonerifolium*, and *Filifolium sibiricum*.

The grazing platform facilities have six stocking rates, which were established in 2009. Each stocking rate has three replicates (Figure 2). The six stocking rates are marked as 0, 1, 2, 3, 4, and 5. These represent the grazing of 0, 2, 3, 4, 6, and 8 heads of 250–300 kg young cattle in each replicate subplot of the six stocking rate treatments, respectively. Grazing activities last for 120 days (between June and October) annually (since 2009). Consequently, the biophysical parameters—including canopy height, fractional canopy cover, and biomass—in each replicate plot differ [42]. For example, the average canopy height of plots W/M/E5 is only 6 cm, corresponding with the eight heads of cattle; however, the average canopy height of plots W/M/E0 reaches 33.7 cm, corresponding to the 0 heads of cattle. Table 1 shows the summary of average subplot characteristics based on 90 field measurements.

### 2.2. Field Vegetation Measurements

Field measurement was carried out by a calibrated staff from 3 to 4 September 2015, a week following the lidar data acquisition, when the aboveground biomass of this meadow steppe reached its annual maximum and some leaves turned yellow. Ninety 1 × 1 m^2^ quadrats (Figure 2) were randomly set in the study area following a simple line point transect method [20], with each grazing subplot having five quadrats. The quadrats within each subplot appear to be clumped, but the minimal distances between the quadrats are longer than 10 m. To reduce the influence of the topographic variation on the paring of the field-measured and lidar-derived heights, each sampling quadrat was located in a homogeneous area in terms of canopy height and fractional cover and the topographic variation within the quadrat was less than 5 cm (i.e., no hollows, depressions, etc.). The measured parameters include the canopy height, fractional cover, biomass, and geographic coordinates of the centers for each quadrat.

**Figure 3:**
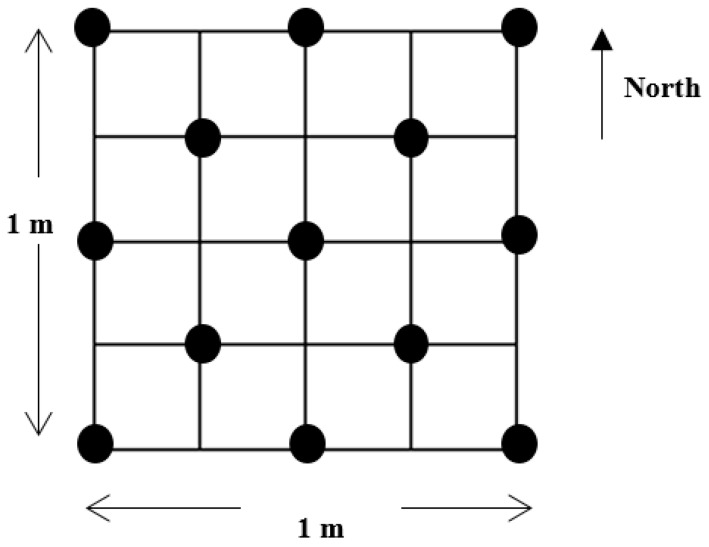
Measurement scheme for canopy height. Thirteen locations (dark dots) were measured in each quadrat to derive the mean, maximum, and minimum values.

The canopy height was measured using a 13-point measurement scheme with a ruler (Figure 3). The mean, maximum, and minimum values of canopy heights at the 13 points were recorded. The fractional canopy cover of each quadrat was measured by overlaying a net with a 10 cm × 10 cm grid on the quadrat, and was then calculated by dividing the number of grids intercepted with plants by the total number of grids in the net [21]. The aboveground biomass, including green and dead stand plants in each quadrat, was clipped and dried for 48 h at 65 °C to constant weight in an oven to calculate the dry weight [42]. Considering the limited detection capability of discrete-return lidar sensors, we excluded the litter biomass from biomass measurements. The geographic coordinates of the 90 quadrats were collected using Garmin portable GPS, which generated an RMSE value of 2.61 m in the horizontal direction. All quadrat values mentioned were used to compare and assess the ability of UAV lidar data to provide similar metrics.

### 2.3. Light Detection and Ranging (Lidar) Data Collection and Pre-Processing

**Figure 4:**
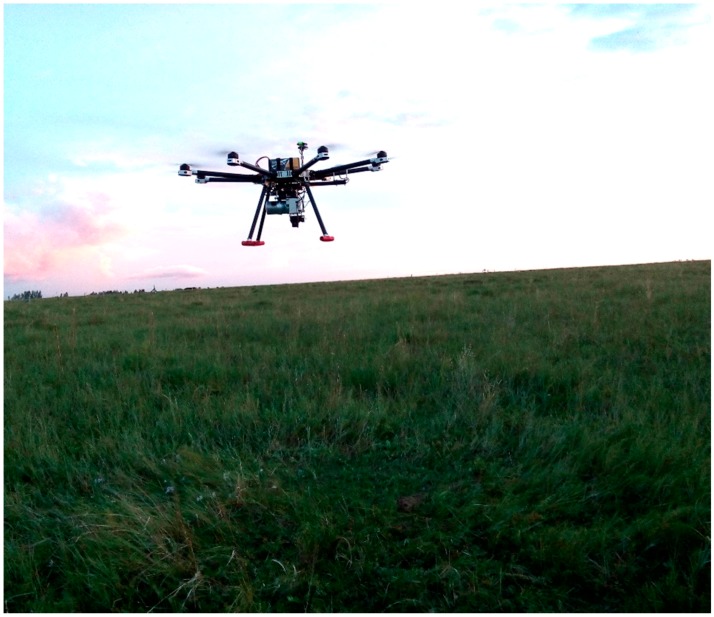
A ground-based photograph indicating the Li-Air fitted with Velodyne’s HDL-32E lidar sensor.

In August 2015, two lidar surveys were flown specifically for use in this study. One survey was collected for direct comparison with field data, with the acquired data covering the entire study area. The UAV platform used in this study was a multi-rotor Li-Air (Figure 4). The flight height was 40 m. The other survey data covering a 50 m × 20 m area (Figure 2) was collected in the subplot E-3 to analyze the influence of flight height on lidar estimates. The flight heights varied from 10 m to 120 m at an interval of 10 m.

The Li-Air has an onboard navigation system based on a navigation-grade GPS receiver and a small microelectromechanical system-based inertial measurement unit (IMU) enabling the Li-Air to fly autonomously through a predefined set of waypoints. The precise coordinates and orientation were determined using a NovAtel SPAN-CPT (Beijing BDStar Navigation Co. Ltd., Beijing, China) global navigation satellite system and IMU. The source of the laser scanning system was the lidar sensor of Velodyne’s HDL-32E (Table 2), which is a 32-beam lidar that weighs only 1 kg. To ensure the accuracy of point clouds, only eight channels with the minimum scan angles were used to derive the canopy indices. The HDL-32E was operated in dual-return mode, i.e., recording the strongest return and the last return, if the distance between the two was greater than 1 m. However, the site consisted of grassland vegetation and bare earth (i.e., no trees, buildings, or other structures); thus, the dataset was almost entirely composed of single-return only pulses. The laser points were geo-referenced to the Universal Transverse Mercator (UTM) coordinate system using the base station GPS and airborne GPS/IMU data, with a horizontal accuracy (RMSE) of better than 1 m [43,44]. The lidar data sampling densities across the area averaged 26 points/m^2^.

### 2.4. Parameter Estimation and Mapping

Three canopy structure indices used in this study were calculated for each quadrat from lidar point clouds, and were then used to estimate biomass. The indices are the mean canopy height (MeanCH, the average of the canopy heights associated with a quadrat), maximum height (MaxCH, the maximum height of the same set of canopy heights), and fractional canopy cover (FVC, the ratio of the number of canopy returns to all returns). Figure 1 outlines the workflow of processing lidar data to generate the canopy height, fractional cover, and biomass maps. Lidar processing routines are similar to those in references [34,39] and are described as follows.

**Figure 5:**
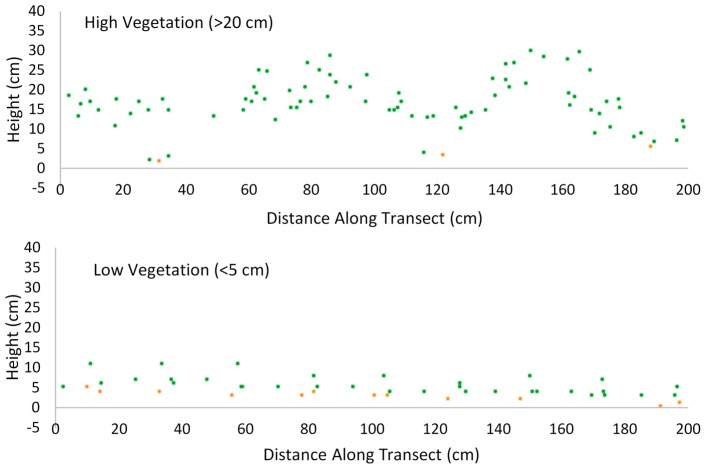
Examples of two grassland canopy transects. The top panel shows data from a high vegetation area. The bottom panel shows data from a low vegetation area. The ground points are shown in yellow color. The canopy points are shown in green color. The transect depth is 0.5 m.

Step 1: Lidar data were classified into ground, vegetation, and other returns by using the slope-and angle-based filtering method [45,46], which has been embedded in the commercial filtering software package of TerraScan (Terrasolid Ltd., Helsinki, Finland) for generation of DEM from lidar data. The classified points were examined to make sure that >90% points were correctly classified. Figure 5 shows two lidar point classification examples from high-vegetation and low-vegetation areas. The *z* coordinate of the vegetation points were rewritten as the relative height from the ground (the vertical distances between the vegetation points and the ground surface model established from the ground point clouds). The relative height point data were gridded without interpolation to establish the canopy height model (CHM), with the *z* value of each 0.2 m cell being the maximum relative height value and the outside *z* value being −0.01. Canopy height metrics were extracted for the 90 sample locations from the CHM. Note: “−0.01” is a marker used to indicate a null *z* value. The cells with *z* values being −0.01 will not be used to calculate the mean, maximum canopy height, and fractional cover metrics in the next step.

Step 2: The mean, maximum canopy height, and fractional cover metrics were calculated for the 90 sample locations. The mean lidar-derived canopy height corresponding to a quadrat with the center coordinates at (i,j) is defined as the mean values of all the pixels within the lidar data window corresponding to the quadrat and is expressed as:

(1) MeanCH(i,j)=Mean[h(u,v)], u,v∈W(i,j)

where h(u,v) indicates the initial canopy height at pixel (u,v). *W* indicates the corresponding lidar data window with the center coordinates at (i,j).

The maximum lidar-derived canopy height corresponding to the quadrat is defined as the maximum values of all the pixels within the *W* and is expressed as:

(2) MaxCH(i,j)=Max[h(u,v)], u,v∈W(i,j)

The fractional vegetation cover corresponding to quadrat (i,j) was calculated by the ratio of the number of canopy returns (>2 cm) to all returns within the *W* and is expressed as:

(3) $$FVC\left(i,j\right)=\frac{N_{h\left(u,v\right)\geq 2\;\mathrm{cm}}}{N_{all}},\;u,v\in W\left(i,j\right)$$

Step 3: Linear and nonlinear regression models were established based on the relationships between the lidar-derived canopy structure indices and the field measurements. The nonlinear regression included logarithmic, power and exponential models. Coefficients of determination (*R*^2^) of the regression models were calculated using a random selection of 66 samples (training samples). The root mean square error (RMSE) and the relative RMSE (rRMSE, %) of the regression models were calculated using the reserved 24 samples (test samples). The RMSE is defined as:

(4) $$\mathrm{RMSE}=\sqrt{\frac{\sum {\left[y_{i}-{\hat{y}}_{i}\right]}^{2}}{N}}$$

where *N* is the number of test samples, $y_{i}$ is the field-measured values of sample *i*, and ${\hat{y}}_{i}$ is the predicted values of sample *i*. The relative RMSE (rRMSE, %) is defined as a percentage of the maximum *y*.

Finally, three regression models with the best performances were selected to map the mean canopy height, fractional cover, and biomass. The canopy height map was predicted based on the relationship between the mean field-measured height and the mean lidar-derived height. The fractional cover map was predicted based on the relationship between the lidar-derived fractional cover and the field-measured fractional cover. The aboveground biomass map was predicted based on the best single regression model between the mean lidar-derived height and biomass.

The prediction results were evaluated at subplot scale by using the stocking rate patterns of the grazing platform. First, visual inspection was carried out to compare the predicted maps and the stocking rate patterns of the grazing platform and determine if the distributions of the lidar estimates are consistent with the stocking rate patterns in each replicate. The six stocking rates set in the grazing platform have a mean difference of 5.4 cm in canopy height and 2.8% in fractional cover. Furthermore, the mean canopy height, fractional cover, and biomass were calculated for each stocking rate using the pixel data from the predicted maps. The mean values were compared with the corresponding stocking rate patterns on a quantitative level to determine the ability of UAV lidar to distinguish the differences between six stocking rates.

### 2.5. Influence of Flight Height

A multiple height lidar survey was conducted in a 50 m × 20 m area covering five quadrats in the subplot E-3 to analyze the influence of flight height on lidar estimates (Figure 2). The mean canopy height, the standard deviation (σ) of the canopy heights, and the fractional cover derived from the lidar data were calculated for each flight height. These values were then compared with the field measurements calculated by averaging the field-measured values within the five quadrats. The flight heights varied from 10 m to 120 m at an interval of 10 m.

The σ of the lidar-derived canopy heights are defined as:

(5) $$\sigma =\sqrt{\frac{\sum {\left[h\left(u,v\right)-\bar{h}\right]}^{2}}{N}},\;u,v\in W$$

where *W* indicates the 50 m × 20 m area, $\bar{h}$ indicates the mean height of the lidar-derived canopy points within the *W*, and *N* is the number of the points within the *W*.

## 3. Results

### 3.1. Analysis of Field Measurements

**Figure 6:**
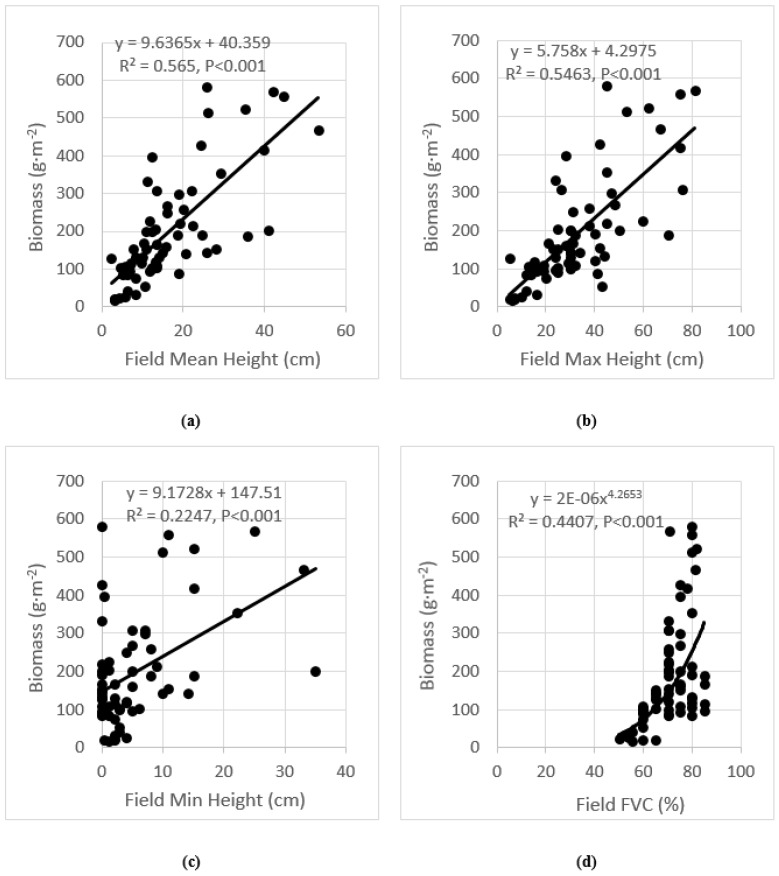
Relationships of biomass with the field-measured mean canopy height (**a**); maximum canopy height (**b**); minimum canopy height (**c**); and fractional cover (**d**). *R*^2^ of regression models were calculated from the 66 training samples.

Linear and nonlinear regression analyses of biomass with the field-collected mean, maximum and minimum heights, as well as fractional cover were first computed for each comparison, displayed in Figure 6, for the 66 training quadrats surveyed. The relationships were examined to determine if there were possible connections between the canopy heights, fractional cover, and the biomass. All correlations reported were significant at *p* < 0.001, and all correlations reported in this study are significant at this level unless stated. In the comparison of all vegetation samples, the mean and maximum canopy height exhibited the strongest correlations with biomass (*R*^2^ = 0.565, and *R*^2^ = 0.562, respectively). The minimum canopy heights showed the lowest correlation of *R*^2^ = 0.2247. The fractional cover showed a correlation of *R*^2^ = 0.4407. The strong correlations showed the potential to estimate the biomass using the canopy heights and fractional cover.

In addition, the results of multiple linear regressions were also promising. It was also observed that when fractional cover was added into the binary linear regressions, the regression of biomass on the mean canopy height was slightly improved from *R*^2^ = 0.565 to 0.590 and RMSE = 95.6 to 94.8 g·m^−2^ (calculated from the 24 independent test samples). This improvement is consistent with previous studies [5,35] and suggests the limited advantages of using both canopy height and fractional cover derived from lidar data to estimate aboveground biomass because of the high correlation between canopy heights and fractional cover. Since field-observed parameters typically have higher accuracy levels than lidar-derived, there is no expectation of stronger correlations existing between biomass and lidar-derived parameters.

### 3.2. Lidar-Derived Parameters

Lidar-derived canopy heights at each subplot followed the expected order, with high vegetation having the tall canopy height and low vegetation having the short canopy height. Figure 6 illustrates examples of the lidar point clouds extracted from a high vegetation area and a low vegetation area. Linear and nonlinear regression analyses were carried out between the three derived-derived canopy structure indices and field-measured indices to determine whether the indices are accurately estimated (Table 3). As listed in Table 3 the lidar consistently underestimated the canopy height, and the lidar-derived fractional cover was obviously different from the field-observed fractional cover (the slope was only 0.228 and the intercept was 54.955). The mean lidar-derived heights were only approximately one-third as high as the mean field-observed heights, although a little overestimation was seen when the canopy height was lower than 5 cm. Further investigation showed that the maximum lidar-derived heights were generally lower than the maximum field-measured heights, but were higher than mean field-measured heights, as expected. This indicated that the ability of lidar to penetrate the grassland canopy is not too bad. However, all the examined lidar-derived parameters showed a moderate to strong relationship with the field-measured parameters, and all these models were statistically significant (*p* < 0.01). This indicated in most cases that lidar-derived indices are not very accurate for grassland ecosystems, but can be calibrated using the field data to predict the actual canopy height and fractional cover due to the relatively high level of correlation.

The relationships of the field-observed biomass with the three-canopy structure indices derived from lidar data were also assessed (Table 4). Although all correlations reported were lower than those listed in Section 3.1 as expected, all correlations reported were significant with *p* < 0.001 and the accuracies (RMSE), calculated by using the 24 independent test datasets, were high with RMSE < 90.2 g·m^−2^. Among the three examined indices, the mean lidar-derived canopy height exhibited the strongest linear correlation (*R*^2^ = 0.34, RMSE = 81.9 g·m^−2^, and relative RMSE = 14.1%) with the aboveground biomass. The fractional cover showed the lowest correlation with the aboveground biomass (*R*^2^ = 0.316, RMSE = 90.18 g·m^−2^, and relative RMSE = 15.5%). The results of multiple regressions were also promising. However, most of the results indicated almost no improvement in comparison with the simple regressions using the lidar-derived mean height. The *R*^2^ and RMSE values were only slightly improved to 0.341 and 81.85 g·m^−2^ (from 0.34 and 81.9 g·m^−2^), respectively. These results are in good agreement with the conclusion drawn from the field-measured data: the combination of the derived mean canopy height and fractional cover could not significantly improve the accuracy of biomass estimation because of the high correlation between lidar-derived mean canopy heights and fractional cover (*r* = 0.958, Table 5). In fact, all the lidar-derived canopy structure indices were highly correlated (*r* > 0.69, as shown in Table 5). As a result, it is less necessary to combine the derived mean canopy height and fractional cover for biomass estimation in this study.

**Figure 7:**
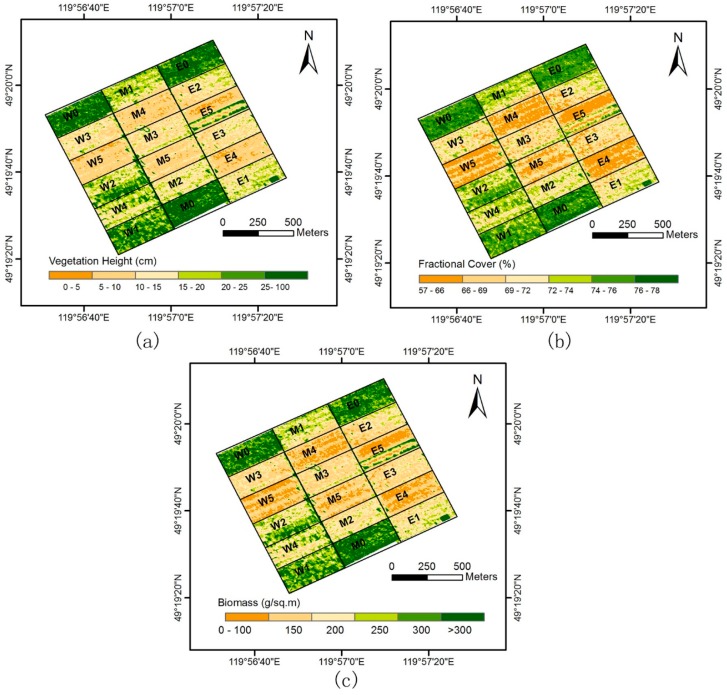
Predicted maps. (**a**) Canopy height map predicted using the mean lidar-derived height; (**b**) Fractional cover map predicted using the lidar-derived fractional cover; (**c**) Aboveground biomass map predicted using the mean lidar-derived height.

**Figure 8:**
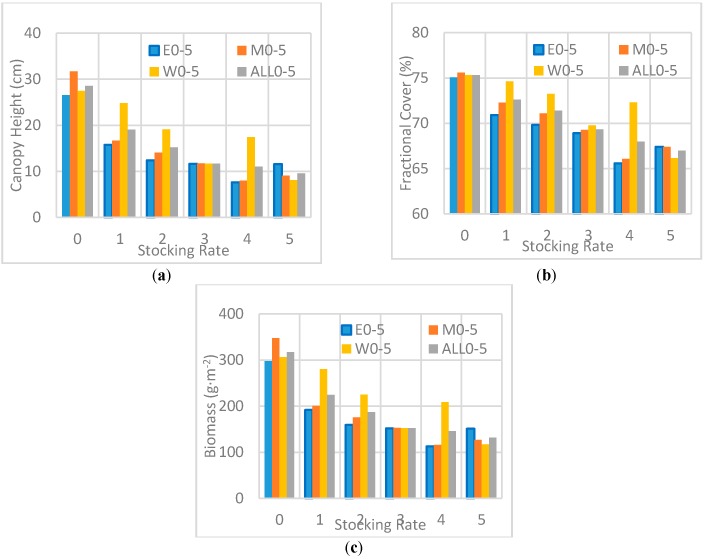
Mean values of the six stocking rates at plot level using pixel data from predicted maps. (**a**) Plot-level mean canopy height of the six stocking rates; (**b**) Plot-level mean fractional cover of the six stocking rates; (**c**) Plot-level mean aboveground biomass of the six stocking rates. Spatial resolution is 1 m. Plot designations E (East), M (Middle), W (West) and ALL (combined data).

Using the prediction models presented shown in Table 3 and Table 4, the canopy height, fractional cover, and biomass maps were generated (Figure 7). The prediction models and results were then evaluated using the stocking rate patterns of the grazing platform and field biomass. Visual inspection showed the distributions of the lidar estimates shown in Figure 7 seemed to be consistent with the stocking rate patterns in each replicate (Figure 2), with low vegetation exhibited in areas of high stocking rates (higher grazing intensity), and high vegetation exhibited in areas of low stocking rates. However, whether the six plot-level stocking rates can be distinguished is difficult to determine. To investigate more closely, we also calculated the mean canopy heights, fractional cover, and biomass for the six stocking rates at plot level using pixel data from the predicted maps (Figure 8). On a quantitative level, the mean values of the six stocking rates at plot level should be representative of a reduction in height, fractional cover, and biomass associated with increasing stocking rate since more cattle had grazed on each subplot.

The mean canopy height, fractional cover, and biomass at All0-5 decreased as stocking rates increased from 0 to 5. However, not every mean estimate was in accord with the decrease trend due to existence of enclosed experimental areas, a data-acquisition problem, and other reasons. For example, obvious overestimates were observed in the stocking rate, W4. It was found that moving from stocking rates W4 to W5 was associated with a move from six to eight cattle; this inability to detect differences at this level of stocking was due to a saturation of the subplot with the yield diminished by the presence of six cattle and not suited to sustain eight. The field measurements based on 88 quadrats only exhibited a difference of 0.8 cm in canopy height between the stocking rates 4 and 5 (Table 1). At this time, the overestimates may have been caused by the topographic change or the measurement error of the Velodyne HDL-32E. The Velodyne HDL-32E specification states a measurement accuracy of ±2 cm. After further visual inspection of Figure 7a, we found a further explanation related to a bright (overestimated) block in subplot E5 where canopy heights were obviously greater than the rest. The canopy heights in the eastern stocking rates, E0-5, did not decrease when stocking rates moved from 4 to 5, which can be explained by the existence of the abnormal data block in E5, where the canopy heights are obviously higher than the rest and increased the average heights of the subplots. Overall, the employed discrete-return lidar has the potential to distinguish the six stocking rates.

### 3.3. Influence of Flight Height

**Figure 9:**
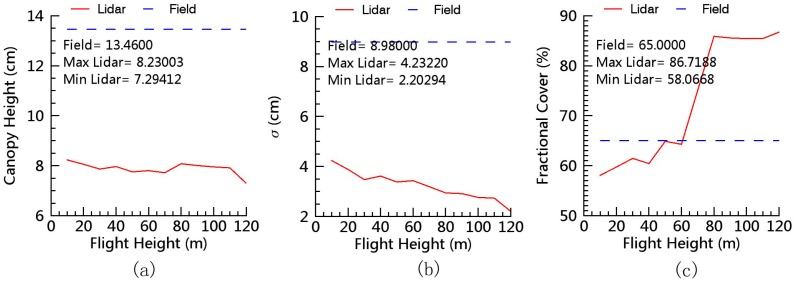
Influence of flight height on lidar-derived mean canopy height (**a**); RMSE of the canopy height (**b**); and fraction canopy cover (**c**). Field/Lidar σ: the standard deviation of field measured/lidar-derived canopy heights.

We also analyzed the influence of flight height on the estimation of grassland canopy heights and fractional cover (Figure 9). In the figure, both the standard deviation (RMSE) and mean of the lidar-derived canopy heights were on downward trends over the flight height. The mean lidar-derived canopy height decreased by 0.94 cm (from 8.23 cm to 7.29 cm), but the RMSE of lidar-derived canopy height deceased 2.03 cm (from 4.23 cm to 2.20 cm), and the lidar-derived canopy cover increased from 58.1% to 86.7% as the flight height increased from 10 m to 120 m. The fast decrease of the σ and fractional cover show that flight height has a significant influence on the detailed information because lidar can obtain more returns from the ground and canopy when the flight height is low (the laser footprint size is small). The slow decrease of the mean lidar-derived canopy heights shows that Velodyne HDL-32E has a high accuracy when the flight height is within the range. Note that the official claim of the effective detection range is 100 m, but we still obtained a few returns from the ground and vegetation when flight height was 120 m. In addition, the canopy height and the σ were significantly underestimated at all flight heights, and the fractional cover was significantly overestimated when the flight height was higher than 60 m. For example, the mean field-observed canopy height was approximately twice as high as the mean lidar estimate. To obtain high-detail data, the flight height was set to 40 m in this study to collect data for the entire study area.

## 4. Discussion

### 4.1. Advantages

This study demonstrated the feasibility of deriving the grassland canopy height, fractional cover, and biomass using a smart UAV discrete lidar and a simple methodology to improve effectively the accuracy of lidar estimates. This capability was examined by comparing the ground-based measurements and lidar estimates. The results of this study suggested that several lidar-derived canopy structure indices correlated well with the field-measured parameters (Table 3 and Table 4). The lidar-derived heights and fractional cover also correlated well with the field-measured biomass. The mean lidar-derived canopy height exhibited the strongest linear correlation (*R*^2^ = 0.340, RMSE = 81.89 g·m^−2^, and relative error of 14.1%) with the aboveground biomass among the examined canopy structure indices. The findings also suggested the limited improvement of the *R*^2^ and RMSE values when including an additional canopy index. The *R*^2^ and RMSE values were slightly improved to 0.341 and 81.85 g·m^−2^, respectively, when canopy height and fractional cover were added in the regression. The reason for the limited improvement is that lidar-derived canopy height is highly correlated to fractional cover, with an *r* of 0.958 (Table 5). Other researchers working with different vegetation species, including some in forests and shrubs, also found similar relationships [35,47,48,49]. As expected in Section 3.1, the *R*^2^ values between the lidar-derived metrics and biomass were lower than those between the field measurements and biomass. Nevertheless, our results are comparable to those for salt marsh when full waveform lidar is used [5]. The agreements is encouraging given the GPS positioning accuracy and the time differences between the quadrat and lidar surveys (a week of cattle-grazing activities may slightly change the canopy height and biomass patterns).

Stocking rate patterns of the cattle-grazing platform were also used to self-validate the lidar estimates in addition to the field observed data. Figure 7 shows that the lidar-derived canopy height, fractional cover and biomass distributions are roughly consistent with the stocking rate patterns shown in Figure 2. High stocking rate subplots have low vegetation and low stocking rate subplots have high vegetation. Lidar-derived parameters can also distinguish between the six stocking rate treatments set in the study area, as statistical differences were exhibited between stocking rates. The high coincidence between lidar estimates and stocking rate patterns also shows that discrete-return lidar, such as Velodyne HDL-32E, is a highly promising tool for estimating vegetation height and biomass in grassland ecosystems. Furthermore, compared with optical and SAR remote sensing, the examined UAV discrete lidar also has the advantages of monitoring the grasslands with the following two characteristics:

(1) The changes in canopy height or cover are remarkable. Previous researchers have reported that VI-based optical remote sensing has a severe saturation problem on the estimation of biomass in grasslands with high canopy cover or leaf area index because of the limited capability of detecting canopy height [2,21]. Backscatter-based SAR sensors are less sensitive to the change in canopy structure parameters and biomass for short vegetation, such as grasses [28,29]. However, lidar can directly measure the three-dimensional aspects of vegetation canopy. A combination of lidar-derived canopy height and fractional cover has been shown to accurately estimate aboveground biomass even in high biomass ecosystems, where passive optical and active microwave sensors typically suffer saturation problems [33,34]. This study also demonstrates a high accuracy for estimating the biomass with the mean canopy height varying from 6 cm to 33.7 cm (Figure 7 and Table 4).
(2) A significant amount of dead vegetation exists (e.g., the grassland during non-growing seasons). VI-based optical and backscatter-based SAR sensors depend heavily on vegetation chlorophyll and water contents [2,20], and exhibit high uncertainty in monitoring the non-growing season vegetation [50]. However, lidar discrete-return range data is independent from vegetation chlorophyll and water content, because the lidar sensor itself illuminates the objects and can obtain directly the 3D canopy structure information. Consequently, lidar data is the most suitable remotely sensed data for measuring canopy structure parameters and biomass regardless of the vegetation is alive or dead. This is very important for grassland managers to monitor the non-growing season grasslands, because non-growing seasons typically account for nearly three-quarters of a year [42], and the forage remaining is the main food source for livestock and wild animals to last the non-growing seasons.

### 4.2. Error Sources

Despite the numerous advantages demonstrated, almost all the canopy heights and most of the fractional covers were underestimated from the lidar data. Similar conclusions were drawn for taller species associated with forests and shrubs [33]. Such a finding can be attributed to the vegetative characteristics of different species [5], capability of discrete-return lidars to penetrate grassland canopy, limitation in obtaining returns from the ground, and introduction of significant topographic relief to the lidar-derived canopy heights. The main source of the underestimates in high canopy subplots and overestimates in low canopy subplots is related to the vegetative characteristics of different species. For example, *P. turczaninovii* are typically much lower than *S. baicalensis*, but the mean biomass is typically double or higher than that of *S. baicalensis*. This is also related to the large laser beam divergence and the long transmit pulse width [5,44].

Another source of unexplained variance may be related to flight height, which is positively correlated to lidar footprint size, thereby determining the capability of discrete-return lidars to penetrate grassland canopy [34]. Figure 9 shows that the influence of flight height on grassland canopy height estimates is small, but the influence of flight height on the derived fractional cover and the detailed data is obvious. The mean lidar-derived canopy heights decreased by only 0.94 cm, the RMSE of lidar-derived canopy heights deceased by 2.03 cm, and the lidar-derived canopy cover increased from 58.1% to 86.7%, as flight height increased from 10 m to 120 m. These results are consistent with those reported by previous studies on forests [34,51,52]. Therefore, low flight height is required when highly detailed data are needed. The recommended flight height is 40–60 m for the typical meadow-steppe grassland with a fractional cover of ~65%. Further research is needed to investigate the optimal flight height suitable for grasslands with different fractional cover.

The non-coincidence of the geopositional information from field quadrat centers and lidar-derived metric centers also has a negative influence on the accuracy of lidar estimates [53,54,55]. The hand-held GPS gave a positional accuracy of 2.61 m, while the quadrat size was only 1 m × 1 m. It is impossible to improve significantly the correlation coefficients between ground and remote sensing metrics, although the accuracy and resolution of lidar data used in this study are very high (the accuracy is better than 1 m, and the resolution is 20 cm). This highlights the need of high-accuracy ground GPS measurements, such as real time kinetic (RTK) global positioning system (GPS) systems, for grassland study, where the quadrat size is generally smaller than 1 m × 1 m because of cost limits.

## 5. Conclusions

A light unmanned aerial vehicle (UAV)-based discrete-return light detection and ranging (lidar) system was tested for extracting both the canopy height and fractional cover, which were then used to estimate the aboveground biomass. The primary conclusions from this study can be summarized as follows: (1) The Velodyne HDL-32E can yield vital returns for estimating grassland canopy height, fractional cover, and biomass and can provide an alternative to field-based data collection. Among the three canopy structure indices examined, mean canopy height is the best predictor of aboveground biomass and explains the greatest proportion of variance (*R*^2^ = 0.340, RMSE = 81.89 g·m^−2^, and relative error of 14.1%). It is unnecessary to establish multiple regressions by adding the fractional cover to improve the *R*^2^ and RMSE values since the correlation between mean canopy height and fractional cover is very high in this study; (2) The discrete-return lidar alone would underestimate grassland canopy height, and calibration using field data is required to achieve a centimeter-level accuracy; (3) Flight height has no significant influence on the derived canopy height, but has a significant influence on the derived fractional cover and details of the lidar data.

UAV fitted with a discrete-return lidar, such as Velodyne HDL-32E, holds considerable potential to provide highly accurate grassland vegetation measurements, such as canopy height, fractional cover, and biomass, on a large scale (1–10 km^2^) [5,53,56]. Furthermore, UAV discrete lidar is independent on vegetation chlorophyll and water contents. Thus, it has an obvious advantage in monitoring non-growing season grasslands with a large amount of dead vegetation compared with optical and Synthetic Aperture Radar (SAR) remote sensing. However, the methods presented in this study should be further tested. The use of the examined lidar sensor was limited by the large laser beam divergence (the laser footprint size reaches 0.12 m at 40 m flight attitude). This divergence served to reduce the number of returns from ground and canopy top in many cases. Our future studies aim to assess the accuracy of lidar systems with shorter transmit pulse widths and smaller laser beam divergences in estimation of grassland parameters.

## Figures and Tables

**Table 1:** Summary of average subplot characteristics.

Subplot No.	Mean Canopy Height (cm)		Mean Fractional Cover (%)		Mean Biomass (g·m^−2^)	
	Subplot Level	Grazing Rate Level	Subplot Level	Grazing Rate Level	Subplot Level	Grazing Rate Level
W0	38.0	33.7	78.6	76.5	528.2	391.2
M0	30.4		74		180.0	
W0	32.7		76.8		465.3	
E1	27.3	21.2	81	75	195.7	211.8
M1	20.5		73		299.2	
W1	16.0		71		140.5	
E2	13.6	12.2	72	77	134.0	144.7
M2	10.6		81		150.5	
W2	12.5		78		149.7	
E3	13.5	13.7	68	69.7	180.6	239.2
M3	16.4		70		225.1	
W3	11.3		71		312.0	
E4	5.2	6.8	69	63.7	121.1	101.9
M4	4.9		58		83.2	
W4	10.2		64		101.5	
E5	8.2	6.0	57.6	62.4	59.7	56.0
M5	4.9		57.6		28.6	
W5	4.9		72		82.7	

**Table 2:** Flight parameters of Velodyne HDL-32E lidar dataset.

Flight Parameter	Value
Flying speed	5 m/s
Flight height	10–120 m at intervals of 10 m (survey #1)
	40 m (survey #2)
Laser beam divergence	3 mrad
Wavelength	903 nm
Rotation rate	5 Hz
Scan angle	+10° to −30° (+5° to −5° remained)
Point density	26 pts/m^2^
Laser footprint	0.12 m (40 m range)

**Table 3:** Results of linear and nonlinear regression analyses between the lidar-derived parameters and field-measured parameters.

Independent Variable (*x*)	Dependent Variable (*y*)	Model	*R*^2^	RMSE	rRMSE (%)	*p*
Lidar mean height	Field mean height	*y* = 3.557*x* − 3.167	0.583	4.9 cm	9.4	<0.001
Lidar max height	Field max height	*y* = 0.801*x* + 14.74	0.201	10.6 cm	13.1	<0.001
Lidar max height	Field mean height	*y* = 0.660*x* + 0.9376	0.368	17.1 cm	32.2	<0.001
Lidar fractional cover	Field fractional cover	*y* = 0.228*x* + 54.955	0.206	4.4%	5.2	<0.001

Note: Coefficients of determination (*R*^2^) of the regression models were calculated from the 66 training samples and the RMSE (g·m^−2^) were calculated from the 24 independent test samples. The relative RMSE (rRMSE, %) is a percentage of the maximum *y*.

**Table 4:** Linear and nonlinear regression analyses between the lidar-derived parameters and the field-measured biomass.

Independent Variable (*x*)	Model	*R*^2^	RMSE (g·m^−2^)	rRMSE (%)	*p*
**Lidar mean height**	***y* = 34.785*x* + 7.081**	**0.340**	**81.89**	**14.1%**	**<0.001**
Lidar max height	*y* = 5.522*x* + 68.397	0.157	87.96	15.1%	<0.001
Lidar FVC	*y* = 22.842e^0.0266*x*^	0.316	90.18	15.5%	<0.001
**Lidar mean height (*x*_1_) + Lidar FVC (*x*_2_)**	***y* = 35.087*x*_1_ − 0.0467*x*_2_ + 8.711**	**0.341**	**81.85**	**14.06%**	**<0.001**
Lidar mean height (*x*_1_) + Lidar max height (*x*_2_)	*y* = 37.799*x*_1_ − 0.958*x*_2_ − 12.679	0.342	87.93	15.1%	<0.001
Lidar max height (*x*_1_) × Lidar FVC (*x*_2_)	*y* = 1.046*x*_1_ + 3.927*x*_2_ − 102.860	0.276	82.07	14.1%	<0.001

Note: Coefficients of determination (*R*^2^) of regression models were calculated from the 66 training samples, the RMSE (g·m^−2^) were calculated from the 24 independent test samples. The relative RMSE (rRMSE, %) is a percentage of the maximum field-observed biomass (582.5 g·m^−2^). Bold data are the results of the best single and multiple regression models.

**Table 5:** Pearson’s correlations (*r*) between the lidar-derived canopy indices.

	Lidar-Derived Mean Height	Lidar-Derived Max Height	Lidar-Derived FVC
Lidar-derived mean height	1	-	-
Lidar-derived max height	0.760	1	-
Lidar-derived FVC	0.9584	0.696	1

## References

[1] Rango A., Laliberte A., Herrick J.E., Winters C., Havstad K., Steele C., Browning D. (2009). Unmanned aerial vehicle-based remote sensing for rangeland assessment, monitoring, and management. J. Appl. Remote Sens..

[2] Jin Y., Yang X., Qiu J., Li J., Gao T., Wu Q., Zhao F., Ma H., Yu H., Xu B. (2014). Remote sensing-based biomass estimation and its spatio-temporal variations in temperate grassland, Northern China. Remote Sens..

[3] Feng X., Fu B., Lu N., Zeng Y., Wu B. (2013). How ecological restoration alters ecosystem services: An analysis of carbon sequestration in China’s loess plateau. Sci. Rep..

[4] Brown L. (1989). Grasslands. The Audubon Society Nature Guides.

[5] Rogers J.N., Parrish C.E., Ward L.G., Burdick D.M. (2015). Evaluation of field-measured vertical obscuration and full waveform lidar to assess salt marsh vegetation biophysical parameters. Remote Sens. Environ..

[6] Li F., Zeng Y., Luo J., Ma R., Wu B. (2016). Modeling grassland aboveground biomass using a pure vegetation index. Ecol. Indic..

[7] Okin G.S. (2008). A new model of wind erosion in the presence of vegetation. J. Geophys. Res. Atmos..

[8] Fan J., Zhong H., Harris W., Yu G., Wang S., Hu Z., Yue Y. (2008). Carbon storage in the grasslands of China based on field measurements of above- and below-ground biomass. Clim. Chang..

[9] Ni J. (2004). Estimating net primary productivity of grasslands from field biomass measurements in temperate Northern China. Plant Ecol..

[10] Azzari G., Goulden M.L., Rusu R.B. (2013). Rapid characterization of vegetation structure with a microsoft kinect sensor. Sensors.

[11] Richardson J.J., Moskal L.M., Bakker J.D. (2014). Terrestrial laser scanning for vegetation sampling. Sensors.

[12] Sakowska K., Gianelle D., Zaldei A., MacArthur A., Carotenuto F., Miglietta F., Zampedri R., Cavagna M., Vescovo L. (2015). Whiteref: A new tower-based hyperspectral system for continuous reflectance measurements. Sensors.

[13] Jia K., Liang S., Gu X., Baret F., Wei X., Wang X., Yao Y., Yang L., Li Y. (2016). Fractional vegetation cover estimation algorithm for Chinese GF-1 wide field view data. Remote Sens. Environ..

[14] Jia W., Liu M., Yang Y., He H., Zhu X., Yang F., Yin C., Xiang W. (2016). Estimation and uncertainty analyses of grassland biomass in Northern China: Comparison of multiple remote sensing data sources and modeling approaches. Ecol. Indic..

[15] Xu D., Guo X. (2015). Some insights on grassland health assessment based on remote sensing. Sensors.

[16] Piao S., Fang J., Zhou L., Tan K., Tao S. (2007). Changes in biomass carbon stocks in China’s grasslands between 1982 and 1999. Glob. Biogeochem. Cycles.

[17] Hill M.J., Donald G.E., Vickery P.J. (1999). Relating radar backscatter to biophysical properties of temperate perennial grassland. Remote Sens. Environ..

[18] Marabel M., Alvarez-Taboada F. (2013). Spectroscopic determination of aboveground biomass in grasslands using spectral transformations, support vector machine and partial least squares regression. Sensors.

[19] Scotta F.C., da Fonseca E.L. (2015). Multiscale trend analysis for pampa grasslands using ground data and vegetation sensor imagery. Sensors.

[20] Gillan J.K., Karl J.W., Duniway M., Elaksher A. (2014). Modeling vegetation heights from high resolution stereo aerial photography: An application for broad-scale rangeland monitoring. J. Environ. Manag..

[21] Chen J., Gu S., Shen M., Tang Y., Matsushita B. (2009). Estimating aboveground biomass of grassland having a high canopy cover: An exploratory analysis of in situ hyperspectral data. Int. J. Remote Sens..

[22] Ni W., Ranson K.J., Zhang Z., Sun G. (2014). Features of point clouds synthesized from multi-view ALOS/PRISM data and comparisons with LiDAR data in forested areas. Remote Sens. Environ..

[23] Balzter H., Rowland C.S., Saich P. (2007). Forest canopy height and carbon estimation at monks wood national nature reserve, UK, using dual-wavelength sar interferometry. Remote Sens. Environ..

[24] Cartus O., Santoro M., Kellndorfer J. (2012). Mapping forest aboveground biomass in the northeastern united states with alos palsar dual-polarization L-band. Remote Sens. Environ..

[25] Renaudin E., Mercer B. Forest biomass derivation from single pass dual baseline polarisation coherence tomography. Proceedings of the 2012 IEEE International Geoscience and Remote Sensing Symposium (IGARSS).

[26] Cloude S.R. (2006). Polarization coherence tomography. Radio Sci..

[27] Michelakis D., Stuart N., Brolly M., Woodhouse I.H., Lopez G., Linares V. (2015). Estimation of woody biomass of pine savanna woodlands from ALOS PALSAR imagery. IEEE J. Sel. Top. Appl. Earth Observ. Remote Sens..

[28] Voormansik K., Jagdhuber T., Olesk A., Hajnsek I., Papathanassiou K.P. (2013). Towards a detection of grassland cutting practices with dual polarimetric terrasar-X data. Int. J. Remote Sens..

[29] Schuster C., Ali I., Lohmann P., Frick A., Forster M., Kleinschmit B. (2012). Towards detecting swath events in terrasar-X time series to establish natura 2000 grassland habitat swath management as monitoring parameter. Remote Sens..

[30] Lee Y.-K., Park J.-W., Choi J.-K., Oh Y., Won J.-S. (2012). Potential uses of terrasar-X for mapping herbaceous halophytes over salt marsh and tidal flats. Estuar. Coast. Shelf Sci..

[31] Mitchell J.J., Shrestha R., Spaete L.P., Glenn N.F. (2015). Combining airborne hyperspectral and lidar data across local sites for upscaling shrubland structural information: Lessons for hyspiri. Remote Sens. Environ..

[32] Olsoy P.J., Glenn N.F., Clark P.E. (2014). Estimating sagebrush biomass using terrestrial laser scanning. Rangel. Ecol. Manag..

[33] Gwenzi D., Lefsky M.A. (2014). Modeling canopy height in a savanna ecosystem using spacebome lidar waveforms. Remote Sens. Environ..

[34] Wasser L., Day R., Chasmer L., Taylor A. (2013). Influence of vegetation structure on lidar-derived canopy height and fractional cover in forested riparian buffers during leaf-off and leaf-on conditions. PLoS ONE.

[35] Lefsky M.A., Cohen W.B., Harding D.J., Parker G.G., Acker S.A., Gower S.T. (2002). Lidar remote sensing of above-ground biomass in three biomes. Glob. Ecol. Biogeogr..

[36] Reddy A.D., Hawbaker T.J., Wurster F., Zhu Z., Ward S., Newcomb D., Murray R. (2015). Quantifying soil carbon loss and uncertainty from a peatland wildfire using multi-temporal lidar. Remote Sens. Environ..

[37] Lefsky M.A. (2010). A global forest canopy height map from the moderate resolution imaging spectroradiometer and the geoscience laser altimeter system. Geophys. Res. Lett..

[38] Dubayah R.O., Sheldon S.L., Clark D.B., Hofton M.A., Blair J.B., Hurtt G.C., Chazdon R.L. (2010). Estimation of tropical forest height and biomass dynamics using lidar remote sensing at La Selva, Costa Rica. J. Geophys. Res. Biogeosci..

[39] Bork E.W., Su J.G. (2007). Integrating lidar data and multispectral imagery for enhanced classification of rangeland vegetation: A meta analysis. Remote Sens. Environ..

[40] Wallace L., Lucieer A., Watson C.S. (2014). Evaluating tree detection and segmentation routines on very high resolution UAV LiDAR data. IEEE Trans. Geosci. Remote Sens..

[41] Wallace L., Lucieer A., Malenovský Z., Turner D., Vopěnka P. (2016). Assessment of forest structure using two uav techniques: A comparison of airborne laser scanning and structure from motion (SFM) point clouds. Forests.

[42] Yan R., Xin X., Yan Y., Wang X., Zhang B., Yang G., Liu S., Deng Y., Li L. (2015). Impacts of differing grazing rates on canopy structure and species composition in hulunber meadow steppe. Rangel. Ecol. Manag..

[43] Middleton J.H., Cooke C.G., Kearney E.T., Mumford P.J., Mole M.A., Nippard G.J., Rizos C., Splinter K.D., Turner I.L. (2013). Resolution and accuracy of an airborne scanning laser system for beach surveys. J. Atmos. Ocean. Technol..

[44] Tulldahl H.M., Bissmarck F., Larsson H., Grönwall C., Tolt G. Accuracy evaluation of 3D lidar data from small UAV. Proceedings of the SPIE Conference on Electro-Optical Remote Sensing, Photonic Technologies, and Applications IX.

[45] Maas H.G., Vosselman G. (1999). Two algorithms for extracting building models from raw laser altimetry data. ISPRS J. Photogramm. Remote Sens..

[46] Axelsson P. (2000). DEM generation from laser scanner data using adaptive TIN models. Int. Arch. Photogramm. Remote Sens..

[47] Morris J.T., Haskin B. (1990). A 5-yr record of aerial primary production and stand characteristics of spartina alterniflora. Ecology.

[48] Möller I. (2006). Quantifying saltmarsh vegetation and its effect on wave height dissipation: Results from a UK east coast saltmarsh. Estuar. Coast. Shelf Sci..

[49] Ni-Meister W., Lee S., Strahler A.H., Woodcock C.E., Schaaf C., Yao T., Ranson K.J., Sun G., Blair J.B. (2010). Assessing general relationships between aboveground biomass and vegetation structure parameters for improved carbon estimate from lidar remote sensing. J. Geophys. Res. Atmos..

[50] Tagesson T. (2014). Using earth observation-based dry season NDVI trends for assessment of changes in tree cover in the Sahel. Int. J. Remote Sens..

[51] Li F., Cui X., Liu X., Wei A., Wu Y. (2014). Positioning errors analysis on airborne lidar point clouds. Infrared Laser Eng..

[52] Naesset E. (2009). Effects of different sensors, flying altitudes, and pulse repetition frequencies on forest canopy metrics and biophysical stand properties derived from small-footprint airborne laser data. Remote Sens. Environ..

[53] Hladik C., Schalles J., Alber M. (2013). Salt marsh elevation and habitat mapping using hyperspectral and lidar data. Remote Sens. Environ..

[54] Tang H., Dubayah R., Swatantran A., Hofton M., Sheldon S., Clark D.B., Blair B. (2012). Retrieval of vertical LAI profiles over tropical rain forests using waveform lidar at La Selva, Costa Rica. Remote Sens. Environ..

[55] Tang H., Brolly M., Zhao F., Strahler A.H., Schaaf C.L., Ganguly S., Zhang G., Dubayah R. (2014). Deriving and validating Leaf Area Index (LAI) at multiple spatial scales through lidar remote sensing: A case study in Sierra National Forest, CA. Remote Sens. Environ..

[56] Nayegandhi A., Brock J.C., Wright C.W. (2009). Small-footprint, waveform-resolving lidar estimation of submerged and sub-canopy topography in coastal environments. Int. J. Remote Sens..

